# Upregulation of miR-101a Suppresses Chronic Renal Fibrosis by Regulating KDM3A via Blockade of the YAP-TGF-β-Smad Signaling Pathway

**DOI:** 10.1016/j.omtn.2020.01.002

**Published:** 2020-01-16

**Authors:** Hong Ding, Yanyan Xu, Nan Jiang

**Affiliations:** 1 Department of Nephrology, The Forth Affiliated Hospital, China Medical University, Shenyang 110032, Liaoning Province, P.R. China

**Keywords:** chronic renal fibrosis, microRNA-101a, KDM3A, YAP-TGF-β-Smad signaling pathway, enhancer, TGIF1

## Abstract

Renal fibrosis denotes a common complication of diabetic nephropathy and is a predominant cause of end-stage renal disease. Despite the association between microRNAs (miRNAs or miRs) and renal fibrosis, miRNAs have been reported to play a vital role in the development of chronic renal fibrosis. Therefore, the aim of the present study was to investigate the possible function of miR-101a in chronic renal fibrosis. Initially, microarray-based gene expression profiling of renal fibrosis was employed to screen the differentially expressed genes. An *in vivo* mouse model of chronic renal fibrosis induced by a unilateral ureteral obstruction (UUO) and an *in vitro* cell model induced by aristolochic acid (AA) were constructed. miR-101a expression was examined using a fluorescence *in situ* hybridization (FISH) assay and quantitative reverse transcription polymerase chain reaction (qRT-PCR). Then, the interaction between miR-101a and KDM3A was identified using an online website combined with a dual-luciferase reporter assay. Finally, gain- and loss-of-function experiments were conducted to elucidate the effect of miR-101a on the expression of Col1a1, fibronectin, α-smooth muscle actin (α-SMA), and YAP-TGF-β (transforming growth factor β)-Smad signaling pathway-related genes, as well as the degree of renal fibrosis. miR-101a was poorly expressed while KDM3A was robustly induced in chronic renal fibrosis tissues and cells. In addition, miR-101a could target and downregulate KDM3A expression, which led to elevated TGIF1, inhibited expression of Collagen I (Col1a1), fibronectin, α-SMA, YAP1, and TGF-β2 along with the extent of Smad2/3 phosphorylation, as well as delayed renal fibrosis degree. Besides, overexpressed YAP/TGF-β2 or inhibited TGIF1 partially restored the inhibitory effect of miR-101a on chronic renal fibrosis. Taken together, miR-101a could potentially slow down chronic renal fibrosis by the inactivation of the YAP-TGF-β-Smad signaling pathway via KDM3A, highlighting the potential of miR-101a as a therapeutic target for chronic renal fibrosis treatment.

## Introduction

Renal fibrosis, featured by glomerulosclerosis and tubulointerstitial fibrosis, is the final manifestation of chronic kidney diseases (CKDs).^1^ Renal fibrosis and CKD have been shown to affect 10% of the world’s population, where adults above age 70 account for 50% of the affected individuals.^2^ Factors that contribute to renal fibrosis include tubular epithelial-to-mesenchymal transition (EMT), activation of mesangial and fibroblast cells, inflammatory (monocyte, macrophage, and T cell) infiltration, and apoptosis.^3^ In the event of chronic renal fibrosis, the activated interstitial fibroblasts are considered to be the main pathogenic mediators of renal disease.^4^ At present, chronic renal fibrosis is incurable and the incidence of the affected patients has been increasing steadily all around the world.^5^ Therefore, it is important to elucidate the epigenetic mechanisms of chronic renal fibrosis in order to identify novel therapeutic approaches for CKD.

The development and progression of renal fibrosis can be largely affected by epigenetic modifications such as aberrant microRNAs (miRNAs or miRs) or DNA methyltransferase.^6^ It has been previously demonstrated that miR-29c may play a role in improving renal function and reducing the degree of histological fibrosis, suggesting the potential of miR-29c as a novel and noninvasive marker for renal fibrosis.^7^ miR-23a or miR-27a has been documented to possess a potential ability to attenuate renal fibrosis via muscle-kidney crosstalk.^8^ Moreover, miR-101a has been illustrated to exert an anti-fibrotic property through the reduction of cardiac fibroblast proliferation induced by hypoxia via targeting transforming growth factor β receptor type 1 (TGFβRI).^9^ Emerging evidence has found that histone demethylases (KDMs), the key regulators in histone modifications, such as KDM3A, KDM5C, KDM6A, and KDM6B, can promote renal cell carcinoma (RCC) progression via hypoxia-mediated angiogenesis pathways.^10^ Furthermore, it has also been reported that histone demethylase KDM3A can accelerate vascular neointimal hyperplasia in diabetic rats via activation of AGTR1 and ROCK2 signaling pathways.^11^ Deficiency of KDM3A may disrupt colorectal cancer cell growth and migration; however, these can be rescued by YAP1 overexpression.^12^ YAP has been found as a mechanoregulator of the TGF-β/Smad signaling pathway and renal fibrogenesis.^13^ The TGF-β/Smad signaling pathway exerts an important effect on renal fibrosis, while targeting the TGF-β/Smad3 signaling pathway may represent an effective treatment approach for CKD treatment that is associated with renal fibrosis.^14^ Based on the aforementioned information, we hypothesized that miR-101a might affect the development of chronic renal fibrosis through the regulation of histone demethylase KDM3A and the TGF-β/Smad3 signaling pathway. Thus, the present study was conducted with the aim to investigate the mechanism involving the miR-101a/KDM3A/TGF-β/Smad3 axis in chronic renal fibrosis.

## Results

### The Significance of miR-101a in Chronic Renal Fibrosis

**Figure 1:**
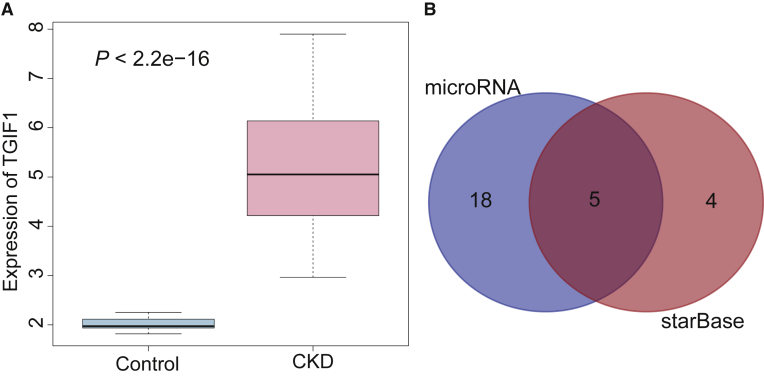
miR-101a Affects Chronic Renal Fibrosis via Regulation of the TGF-β/Smad Signaling Pathway by Targeting KDM3A (A) The expression of TGIF1 in the GEO: GSE66494 dataset in healthy and chronic renal fibrosis samples. The blue box on the left indicates the expression of GEO: GSE66494 in healthy samples, while the red box on the right indicates the expression of GEO: GSE66494 in chronic renal fibrosis samples. (B) Venn diagram demonstrates the upstream miRNAs of KDM3A predicted by microRNA.org and starBase databases, including five intersected miRNAs comprised of mmu-miR-224-5p, mmu-miR-101a-3p, mmu-miR-27a-3p, mmu-miR-144-3p, and mmu-miR-27b-3p.

The signaling pathways associated with renal fibrosis were retrieved from the MalaCards database (https://www.malacards.org) (Table 1). The TGF-β/Smad signaling pathway is a very important signaling pathway of chronic renal fibrosis in mice.^14^ Additionally, the Gene Expression Omnibus (GEO) database revealed that TGFB-induced factor homeobox 1 (TGIF1) of the TGF-β/Smad signaling pathway was found to be differentially expressed in the GEO: GSE66494 dataset, where it was highly expressed in renal fibrosis (Figure 1A). KDM3A is an upstream gene of TGIF1 that can also regulate the TGF-β/Smad signaling pathway via regulation of YAP.^12^^,^^13^ A total of 9 upstream miRNAs of KDM3A were obtained from the starBase database (http://starbase.sysu.edu.cn/), and 23 miRNAs were obtained from the microRNA.org database (http://www.microrna.org/microrna/home.do). The Venn diagram revealed five intersected miRNAs between the two databases (Figure 1B).

**Table 1:** Scores of Pathways Related to Renal Fibrosis

Super Pathways	Score
ERK signaling	13.87
TGF-β pathway	13.49
Akt signaling	13.41
PAK pathway	13.33
Nanog in mammalian ESC pluripotency	13.28

ERK, extracellular signal-regulated kinase; TGF, transforming growth factor; Akt, protein kinase B; PAK, p21-activated kinase; ESC, embryonic stem cell.

Taken together, we hypothesized that miR-101a could target KDM3A-regulated YAP and TGIF1 genes, thereby affecting the TGF-β/Smad signaling pathway and ultimately regulating chronic renal fibrosis.

### miR-101a Is Poorly Expressed in Chronic Renal Fibrosis Tissues and Cells, and Upregulation of miR-101a Attenuates Chronic Renal Fibrosis Progression

**Figure 2:**
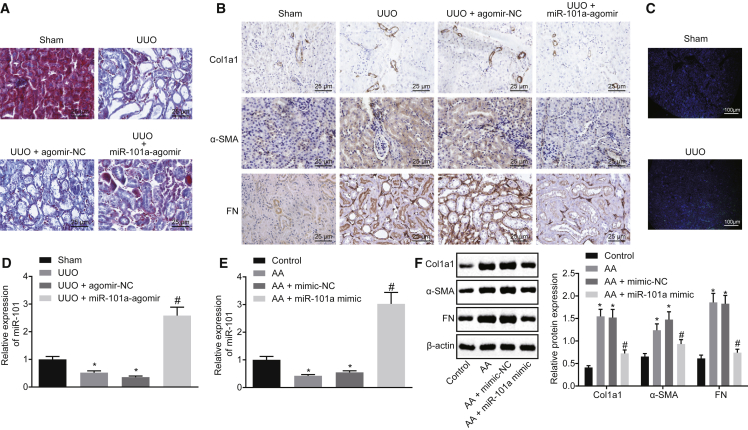
miR-101a Is Poorly Expressed in Chronic Renal Fibrosis Tissues and Cells, and Its Upregulation Delays Chronic Renal Fibrosis (A) Masson’s trichrome staining of kidney tissues of sham-operated and UUO mice (original magnification, ×200). (B) Immunohistochemistry analysis of Col1a1, fibronectin, and α-SMA proteins in kidney tissues of sham-operated and UUO mice (original magnification, ×200). (C) miR-101a expression in kidney tissues of sham-operated and UUO mice determined by FISH (original magnification, ×40). (D) miR-101a expression in kidney tissues of sham-operated and UUO mice determined by qRT-PCR. *p < 0.05 versus sham-operated mice; ^#^p < 0.05 versus mice treated with UUO + agomir-NC. N = 8. (E) miR-101a expression in HK2 cells detected by qRT-PCR. (F) Western blot analysis of Col1a1, fibronectin, and α-SMA proteins in HK2 cells. In (E) and (F), *p < 0.05 versus control cells; ^#^p < 0.05 versus AA-incubated cells transfected with mimic-NC. The above data are all measurement data and expressed as mean ± standard deviation. Data between two groups were compared by an unpaired t test. The experiment was conducted in triplicate.

In order to study the pathological mechanism of renal fibrosis, a chronic renal fibrosis model was constructed *in vivo* using the unilateral ureteral obstruction (UUO) method. Subsequently, Masson’s trichrome staining was conducted to observe the pathological characteristics of UUO mice. The results revealed that UUO mice showed obvious renal fibrosis when compared to sham-operated mice (Figure 2A). Immunohistochemistry detection results revealed a significantly higher positive expression rate of Collagen I (Col1a1), fibronectin, and α-smooth muscle actin (α-SMA) proteins in kidney tissues of UUO mice than that of sham-operated mice (Figure 2B). These findings were indicative of the successful establishment of a mouse model of chronic renal fibrosis.

To investigate the role of miR-101a in renal fibrosis, fluorescence *in situ* hybridization (FISH) and quantitative reverse transcription polymerase chain reaction (qRT-PCR) were employed to detect the expression of miR-101a in kidney tissues. The results demonstrated that the expression of miR-101a in UUO mice was significantly lower than that in sham-operated mice (Figures 2C and 2D). Aristolochic acid (AA) is a well-known fibrogenic molecule, and therefore AA was used to incubate HK2 cell lines cultured *in vitro*. After AA incubation, the expression of miR-101a was notably decreased, while that of Col1a1, fibronectin, and α-SMA was increased clearly, but miR-101a mimic could reverse the effect of AA incubation (Figures 2E and 2F). In addition, UUO mice treated with miR-101a agomir resulted in increased miR-101a expression (Figure 2D), while the positive expression rates of Col1a1, fibronectin, and α-SMA proteins were decreased in the presence of miR-101a agomir (Figure 2B). Furthermore, the results obtained from Masson’s trichrome staining showed that miR-101a-agomir transfection could reduce the fibrosis degree of kidney tissues (Figure 2A). These results concluded that miR-101a was expressed at a low level in UUO-induced chronic renal fibrosis, while the overexpression of miR-101a could prevent chronic renal fibrosis occurrence.

### KDM3A Is Highly Expressed in Chronic Renal Fibrosis and Is a Target Gene of miR-101a

**Figure 3:**
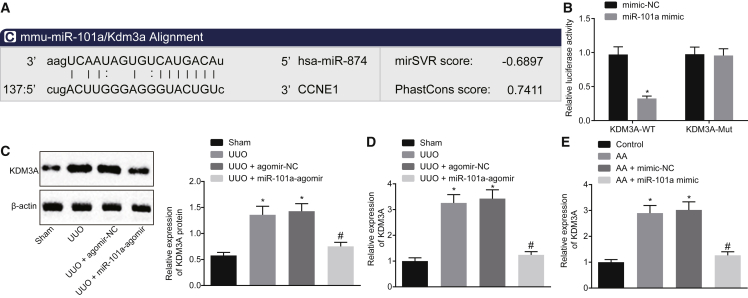
KDM3A Is Highly Expressed in Chronic Renal Fibrosis and miR-101a Targets and Negatively Regulates the Expression of KDM3A (A) Targeting binding sequence between miR-101a and KDM3A analyzed using an online website. CCNE1, G_1_/S-specific cyclin E1. (B) The binding of miR-101a to KDM3A confirmed by a dual-luciferase reporter assay. (C) Western blot analysis of KDM3A protein in UUO mice injected with miR-101a agomir. (D) KDM3A expression in UUO mice injected with miR-101a agomir determined by qRT-PCR. (E) KDM3A expression in AA-incubated cells transfected with miR-101a mimic detected by qRT-PCR. In (B) and (C), *p < 0.05 versus sham-operated mice; ^#^p < 0.05 versus mice treated with UUO + agomir-NC. In (D) and (E), *p < 0.05 versus control cells; ^#^p < 0.05 versus AA-incubated cells transfected with mimic-NC. The above data were all measurement data and are expressed as mean ± standard deviation. Data between two groups were compared by an unpaired t test. The cell experiment was conducted in triplicate.

To clarify the downstream target genes of miR-101a, we applied an online website http://www.microrna.org/ to analyze the possible target genes. The results revealed that there was a targeting binding sequence between miR-101a and KDM3A (Figure 3A). The results of the dual-luciferase reporter assay showed that the luciferase activity of KDM3A-wild-type (WT) could be inhibited by miR-101a mimic, while the luciferase activity of KDM3A mutant (MUT) remained unaffected (Figure 3B). Moreover, studies have shown that KDM3A could regulate the activation of hepatic stellate cells and liver fibrosis through epigenetic regulation of PPARγ. The activation of KDM3A possesses the ability to stimulate the transcription of Timp1. However, as an inhibitor of pan-KDM, JIB-04 could inhibit the left ventricular hypertrophy and fibrosis induced by pressure overload.^15^^,^^16^ We hypothesized that miR-101a may function in chronic renal fibrosis by targeting KDM3A. Therefore, qRT-PCR was employed to detect KDM3A expression in mice with chronic renal fibrosis, with the results demonstrating that KDM3A expression was increased after UUO treatment, but decreased after treatment with miR-101a agomir (Figures 3C and 3D). In addition, KDM3A expression was also increased in AA-incubated HK2 cells but decreased upon miR-101a mimic transfection (Figure 3E). These results suggested that KDM3A was highly expressed in chronic renal fibrosis, while miR-101a could target KDM3A and negatively regulate its expression.

### miR-101a Prevents Renal Fibrosis by Inhibiting KDM3A Expression

**Figure 4:**
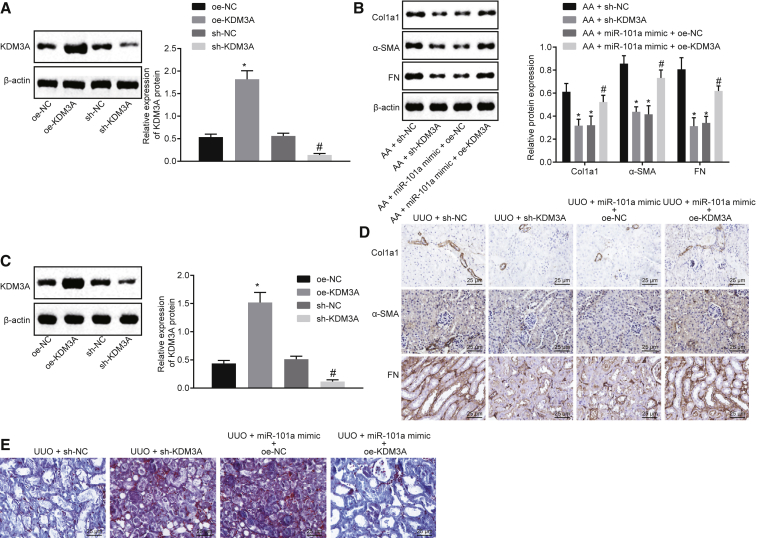
miR-101a Represses Renal Fibrosis via Downregulating the Expression of KDM3A (A) Western blot analysis of KDM3A protein in HK2 cells. *p < 0.05 versus cells transfected with oe-NC; ^#^p < 0.05 versus cells transfected with sh-NC. (B) Western blot analysis of Col1a1, fibronectin, and α-SMA proteins in HK2 cells. *p < 0.05 versus AA-incubated cells transfected with sh-NC; ^#^p < 0.05 versus AA-incubated cells transfected with miR-101a mimic and oe-NC. (C) Western blot analysis of KDM3A protein in kidney tissues of UUO mice. *p < 0.05 versus mice treated with oe-NC; ^#^p < 0.05 versus mice treated with sh-NC. (D) Immunohistochemistry analysis of Col1a1, fibronectin, and α-SMA proteins in kidney tissues of UUO mice (original magnification, ×200). (E) Masson’s trichrome staining of kidney tissues of UUO mice (original magnification, ×200). The above data were all measurement data and are expressed as mean ± standard deviation. Data between two groups were compared by an unpaired t test. N = 8.

In order to elucidate the effect of KDM3A on renal fibrosis, HK2 cells were transfected with short hairpin RNA (sh)-KDM3A and overexpression (oe)-KDM3A. As shown in Figures 4A and 4B, KDM3A expression was elevated in oe-KDM3A-transfected cells while KDM3A expression showed a substantial reduction in sh-KDM3A-transfected cells, in addition to a decline in Col1a1, fibronectin, and α-SMA expression. The expression of Col1a1, fibronectin, and α-SMA protein was upregulated in AA-incubated cells transfected with miR-101a mimic + oe-KDM3A, suggesting that the downregulated KDM3A could significantly decrease the AA-induced upregulation of expression of Col1a1, fibronectin, and α-SMA.

Additionally, the mice injected with lentivirus expressing oe-KDM3A showed increased KDM3A expression while mice injected with lentivirus expressing sh-KDM3A showed downregulated KDM3A expression (Figure 4C). Immunohistochemistry demonstrated that UUO mice treated with sh-KDM3A showed a diminished positive expression rate of Col1a1, fibronectin, and α-SMA in comparison to mice treated with sh-NC, while UUO mice treated with miR-101a agomir + oe-KDM3A showed a decreased positive expression rate of Col1a1, fibronectin, and α-SMA in comparison to mice treated with miR-101a agomir + oe-NC (Figure 4D). Masson’s trichrome staining results showed that knockdown of KDM3A could reduce the fibrosis degree of kidney tissues (Figure 4E). Treatment of miR-101a agomir + oe-KDM3A showed increased fibrosis degree of kidney tissues in UUO mice when compared to miR-101a agomir + oe-NC treatment, indicating that overexpression of KDM3A could reverse the inhibitory effect of upregulated miR-101a on renal fibrosis. Based on the results obtained, we suggested that miR-101a overexpression could inhibit renal fibrosis via suppression of KDM3A.

### KDM3A Promotes TGF-β2 Expression by Regulating the Binding of H3K27ac and TEAD1 to TGF-β2

**Figure 5:**
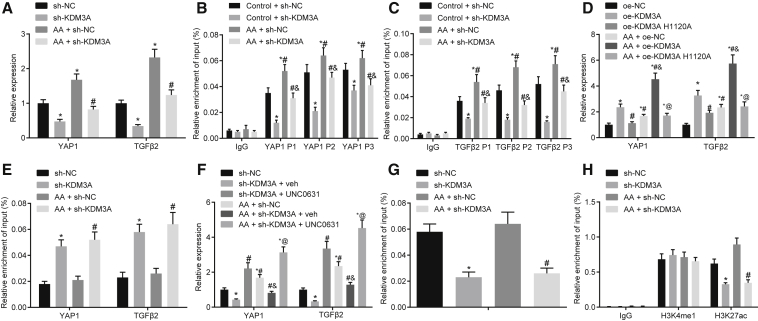
KDM3A Regulates the Expression of YAP1 and TGF-β2 by Altering Enhancer Activity (A) The expression of YAP1 and TGF-β2 upon sh-KDM3A transfection detected by qRT-PCR. *p < 0.05 versus cells transfected with sh-NC; ^#^p < 0.05 versus AA-incubated cells transfected with sh-NC. (B) Enrichment of KDM3A in the promoter region of YAP1 detected by a ChIP assay. (C) Enrichment of KDM3A in the promoter region of TGF-β2 detected by a ChIP assay. In (B) and (C), *p < 0.05 versus the IgG group; ^#^p < 0.05 versus the control + sh-NC group; ^&^p < 0.05 versus AA-incubated cells transfected with sh-NC. (D) The effect of enzymatic activity of KDM3A on YAP1 and TGF-β2 expression detected by qRT-PCR. *p < 0.05 versus cells transfected with oe-NC; ^#^p < 0.05 versus cells transfected with oe-KDM3A; ^&^p < 0.05 versus AA-incubated cells transfected with oe-NC; ^@^p < 0.05 versus AA-incubated cells transfected with oe-KDM3A. (E) The enrichment of H3K9me2 in the promoter region of YAP1 and TGF-β2 detected by a ChIP assay. *p < 0.05 versus cells transfected with sh-NC; ^#^p < 0.05 versus AA-incubated cells transfected with sh-NC. (F) The effect of UNC0631 on YAP1 and TGF-β2 expression detected by qRT-PCR. *p < 0.05 versus cells transfected with sh-NC; ^#^p < 0.05 versus cells transfected with sh-KDM3A and veh; ^&^p < 0.05 versus AA-incubated cells transfected with sh-NC; ^@^p < 0.05 versus AA-incubated cells transfected with sh-KDM3A and veh. (G) Enrichment of H3K27ac and TEAD1 in the promoter region of TGF-β2H detected by a ChIP assay. (H) The enrichment of TEAD1 and H3K27ac in the enhancer of TGF-β2 detected by a ChIP assay. In (G) and (H), *p < 0.05 versus cells transfected with sh-NC; ^#^p < 0.05 versus AA-incubated cells transfected with sh-NC. The above data were all measurement data and are expressed as mean ± standard deviation. Data among multiple groups were analyzed by one-way ANOVA, followed by a Tukey’s multiple comparisons post-test. The cell experiment was conducted in triplicate.

To confirm the involvement of KDM3A in the progression of chronic renal fibrosis by regulating YAP or TGF-β2, the potential effects of KDM3A on the expression of YAP1 and TGF-β2 were examined by qRT-PCR. After knocking down KDM3A in AA-incubated cells, downregulation of YAP1 and TGF-β2 expression was observed (Figure 5A). A chromatin immunoprecipitation (ChIP) assay elaborated that after AA treatment, the enrichment of KDM3A in the promoter regions of YAP1 and TGF-β2 was increased, but it was decreased after knocking down KDM3A (Figures 5B and 5C), implying that the specificity of YAP1 and TGF-β2 was regulated by KDM3A.

KDM3A is known to be the histone demethylase of histone H3 lysine 9 dimethylation (H3K9me2). In order to study whether the regulation of KDM3A in YAP1 expression was associated with the enzymatic activity of KDM3A in HK2 cells, inactivating mutations of KDM3A enzyme, specifically, alanine replacing histidine at amino acid 1120 of KDM3A (H1120A), was performed. When expressed exogenously in HK2 cells, H1120A (inactivating mutations of the histone methyltransferase) showed no effect on the activation of YAP1 and TGF-β2 when compared to KDM3A-WT (Figure 5D), suggesting that KDM3A could regulate the activation of YAP1 and TGF-β2 expression via enzymatic activity of KDM3A. Subsequent results from a ChIP assay revealed that the enrichment of H3K9me2 was elevated after KDM3A knockdown (Figure 5E). Expression of YAP1 and TGF-β2 increased notably upon UNC0631 (an inhibitor of H3K9me2) treatment (Figure 5F). These results suggested that KDM3A may promote the expression of YAP1 and TGF-β2 through H3K9me2.

Recent evidence demonstrated that KDM3A could regulate the binding of H3K27ac and TEAD1 to the enhancer of the Hippo signaling pathway target genes.^12^ To investigate whether the regulation of KDM3A in the expression of TGF-β2 in HK2 cells was related to the recruitment of H3K27ac and TEAD1 in enhancers, a ChIP assay was conducted to detect the enrichment of H3K27ac and TEAD1 in HK2 cells. The results showed that TEAD1 was enriched in the promoter region of TGF-β2 (Figure 5G). Knockdown of KDM3A in AA-incubated cells showed diminished enrichment of TEAD1 and H3K27ac expression in the enhancer of TGF-β2, yet produced no effect on the expression of H3K4me1 (Figure 5H). The aforementioned results suggested that KDM3A may regulate the expression of YAP1 and TGF-β2 through H3K9me2. Moreover, KDM3A might exert a regulatory role on TGF-β2 expression by regulating the binding of H3K27ac and TEAD1 to the enhancer of TGF-β2.

### KDM3A Potentiates Chronic Renal Fibrosis by Activating the YAP-TGF-β-Smad Signaling Pathway

**Figure 6:**
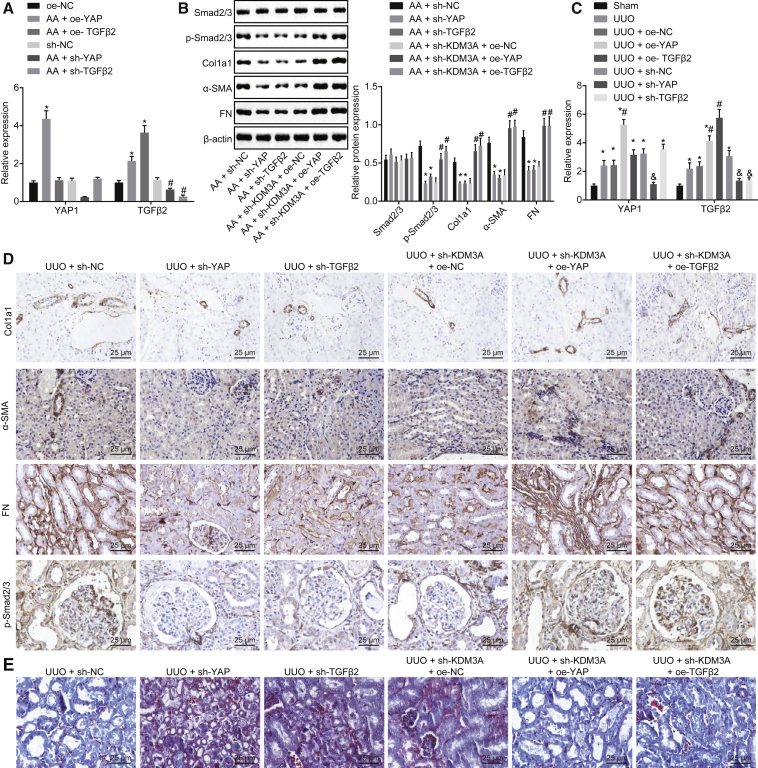
KDM3A Stimulates Chronic Renal Fibrosis via Activation of the YAP-TGF-β-Smad Signaling Pathway (A) The expression of YAP1 and TGF-β2 in cells detected by qRT-PCR. *p < 0.05 versus cells transfected with oe-NC; ^#^p < 0.05 versus cells transfected with sh-NC. (B) Western blot analysis of Col1a1, fibronectin, and α-SMA proteins as well as the extent of Smad2/3 phosphorylation in cells. *p < 0.05 versus AA-incubated cells transfected with sh-NC; ^#^p < 0.05 versus AA-incubated cells transfected with sh-KDM3A and oe-NC. (C) The expression of YAP1 and TGF-β2 in kidney tissues of mice detected by qRT-PCR. *p < 0.05 versus sham-operated mice. # p < 0.05 versus UUO mice treated with oe-NC. (D) Immunohistochemistry analysis of Col1a1, fibronectin, and α-SMA as well as the extent of Smad2/3 phosphorylation in kidney tissues of mice (original magnification, ×200). (E) Masson’s trichrome staining of kidney tissues of mice (original magnification, ×200). The above data were all measurement data and are expressed as mean ± standard deviation. Data among multiple groups were analyzed by one-way ANOVA, followed by a Tukey’s multiple comparisons post-test. The cell experiment was conducted in triplicate.

In order to study whether KDM3A can regulate chronic renal fibrosis through the YAP-TGF-β-Smad signaling pathway, a series of *in vitro* experiments were conducted. Initial results from a qRT-PCR assay showed that the expression of YAP1 was increased in AA-incubated cells transfected with oe-YAP, whereas it decreased upon sh-YAP transfection. The expression of TGF-β2 was promoted in AA-incubated cells transfected with oe-TGF-β2 while it was inhibited after sh-TGF-β2 transfection (Figure 6A), suggesting that YAP1 could regulate the expression of TGF-β, indicating that YAP may be the upstream factor of TGF-β. Subsequent results from western blot analysis showed that AA-incubated cells transfected with sh-YAP and sh-TGF-β2 presented a diminished protein expression of Col1a1, fibronectin, and α-SMA, as well as the extent of Smad2 and Smad3 phosphorylation, while an elevation was detected in the aforementioned factors in AA-incubated cells transfected with sh-KDM3A + oe-YAP or sh-KDM3A + oe-TGF-β2 (Figure 6B).

The expression of YAP1 and TGF-β2 was much higher in UUO mice than that in sham-operated mice. The expression of YAP1 was upregulated in UUO mice treated with oe-YAP, but it was inhibited when treated with sh-YAP. TGF-β2 expression was increased in oe-TGF-β2-treated mice, while it was reduced in the absence of TGF-β2 (Figure 6C). Furthermore, immunohistochemistry results demonstrated decreased positive expression of Col1a1, fibronectin, and α-SMA, as well as inhibited extent of Smad2/3 phosphorylation in UUO mice treated with sh-YAP or sh-TGB-β2; however, treatment with sh-KDM3A + oe-YAP1 or sh-KDM3A + oe-TGF-β2 exhibited a contrasting trend (Figure 6D). Masson’s trichrome staining in Figure 6E showed that the overexpression of YAP1 or TGF-β2 could exacerbate the fibrosis degree of kidney tissues of mice, whereas silencing of YAP1 or TGF-β2 could reverse the tendency. These results suggested that KDM3A could promote chronic renal fibrosis through activation of the YAP-TGF-β-Smad signaling pathway.

### KDM3A Disrupts the Expression of TGIF1, Thus Promoting Chronic Renal Fibrosis

**Figure 7:**
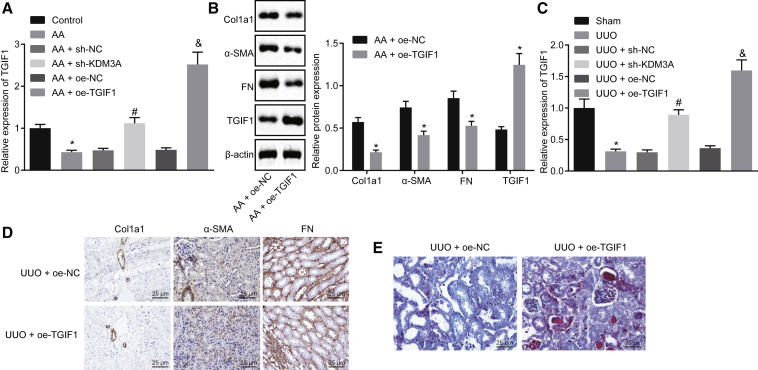
KDM3A Inhibits TGIF1 Expression to Accelerate the Progression of Chronic Renal Fibrosis (A) The expression of TGIF1 in cells assessed by qRT-PCR. *p < 0.05 versus control cells; ^#^p < 0.05 versus AA-incubated cells transfected with sh-NC; ^&^p < 0.05 versus AA-incubated cells transfected with + oe-NC. (B) Western blot analysis of Col1a1, fibronectin, α-SMA, and TGIF1 proteins. *p < 0.05 versus AA-incubated cells transfected with oe-NC. (C) The expression of TGIF1 in kidney tissues of mice assessed by qRT-PCR. *p < 0.05 versus sham-operated mice; ^#^p < 0.05 versus UUO mice treated with sh-NC; ^&^p < 0.05 versus UUO mice treated with oe-NC. (D) Immunohistochemistry analysis of Col1a1, fibronectin, and α-SMA proteins in kidney tissues of mice (original magnification, ×200). (E) Masson’s trichrome staining of kidney tissues of mice (original magnification, ×200). The above data were all measurement data and are expressed as mean ± standard deviation. Data between two groups were compared by an unpaired t test. Data among multiple groups were analyzed by one-way ANOVA, followed by a Tukey’s multiple comparisons post-test. The cell experiment was conducted in triplicate.

The literature has revealed that the interaction between TGIF1 and Smad could inhibit the activity of the TGF-β signaling pathway. In additon, KDM3A has been demonstrated to block the activity of TGIF1^12^^,^^17^. Hence, we hypothesized that KDM3A could directly or indirectly regulate the TGF-β-Smad signaling pathway through YAP/TGF-β2, as well as promote the TGF-β-Smad signaling pathway by inhibiting the expression of TGIF1, thereby regulating chronic renal fibrosis. *In vitro* experiments showed that diminished expression of TGIF1 upon AA treatment was detected, whereas TGIF1 exhibited a notable increase in sh-KDM3A-transfected AA-incubated cells (Figure 7A). In addition, TGIF1 expression was upregulated in AA-incubated cells transfected with oe-TGIF1 (Figures 7A and 7B).

Western blot analysis revealed a decrease in protein expression of Col1a1, fibronectin, and α-SMA in the presence of oe-TGIF1 (Figure 7B). *In vivo* experimental results showed that UUO mice presented with diminished TGIF1 expression in comparison to sham-operated mice, while oe-TGIF1 transfection resulted in elevated TGIF1 expression (Figure 7C). Subsequently, immunohistochemistry analysis demonstrated that UUO mice treated with oe-TGIF1 presented with decreased positive expression of Col1a1, fibronectin, and α-SMA proteins (Figure 7D). Masson’s trichrome staining in Figure 7E showed that the overexpression of TGIF1 could repress the fibrosis of kidney tissues in mice. The aforementioned findings concluded that KDM3A could downregulate the expression of TGIF1 in renal fibrosis, while the overexpression of TGIF1 could repress chronic renal fibrosis.

### Overexpression of YAP/TGF-β2 or Inhibition of TGIF1 Partially Restores the Inhibitory Effect of miR-101a on Chronic Renal Fibrosis

**Figure 8:**
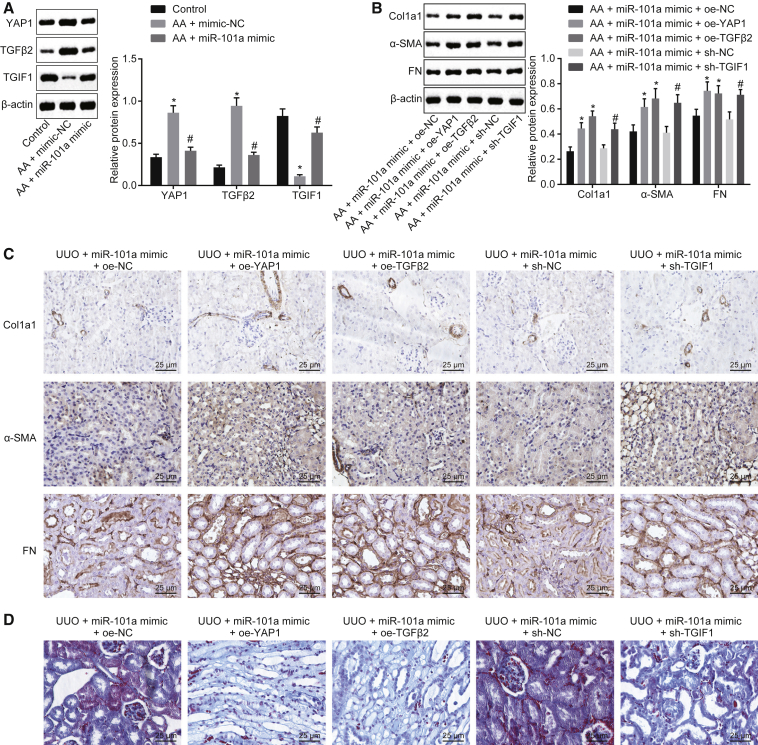
Upregulated YAP/TGF-β2 or Inhibited TGIF1 Facilitates the Inhibitory Effect of miR-101a on Chronic Renal Fibrosis (A) Western blot analysis of YAP1, TGF-β2, and TGIF1 proteins. *p < 0.05 versus control cells; ^#^p < 0.05 versus AA-incubated cells transfected with mimic-NC. (B) Western blot analysis of Col1a1, fibronectin, and α-SMA proteins in cells. *p < 0.05 versus AA-incubated cells transfected with miR-101a mimic and oe-NC; ^#^p < 0.05 versus AA-incubated cells transfected with miR-101a mimic and sh-NC. (C) Immunohistochemistry analysis of Col1a1, fibronectin, and α-SMA proteins in kidney tissues of mice (original magnification, ×200). (D) Masson’s trichrome staining of kidney tissues of mice (original magnification, ×200). The above data were all measurement data and are expressed as mean ± standard deviation. Data among multiple groups were analyzed by one-way ANOVA, followed by a Tukey’s multiple comparisons post-test. The cell experiment was conducted in triplicate.

To investigate the function of miR-101a in chronic renal fibrosis related to the downstream target genes of KDM3A, including YAP1, TGF-β2, and TGIF1, western blot analysis was performed. As illustrated in Figure 8A, AA-incubated cells transfected with miR-101a mimic exhibited a marked reduction in YAP1 and TGF-β2 protein expression, while an increase in TGIF1 expression was observed. The protein expression of Col1a1, fibronectin, and α-SMA was upregulated in AA-incubated cells transfected with miR-101a mimic + oe-YAP1 or miR-101a mimic + oe-TGF-β2, as well as in AA-incubated cells transfected with miR-101a mimic + sh-TGIF1 (Figure 8B). In addition, UUO mice treated with miR-101a agomir + oe-YAP1, miR-101a agomir + oe-TGF-β2, or miR-101a agomir + sh-TGIF1 had upregulated positive expression of Col1a1, fibronectin, and α-SMA proteins, as well as sever fibrosis of kidney tissues of mice (Figures 8C and 8D). Taken together, the overexpressed YAP/TGF-β2 or downregulated TGIF1 could partially aid the inhibitory effect of miR-101a on chronic renal fibrosis.

## Discussion

**Figure 9:**
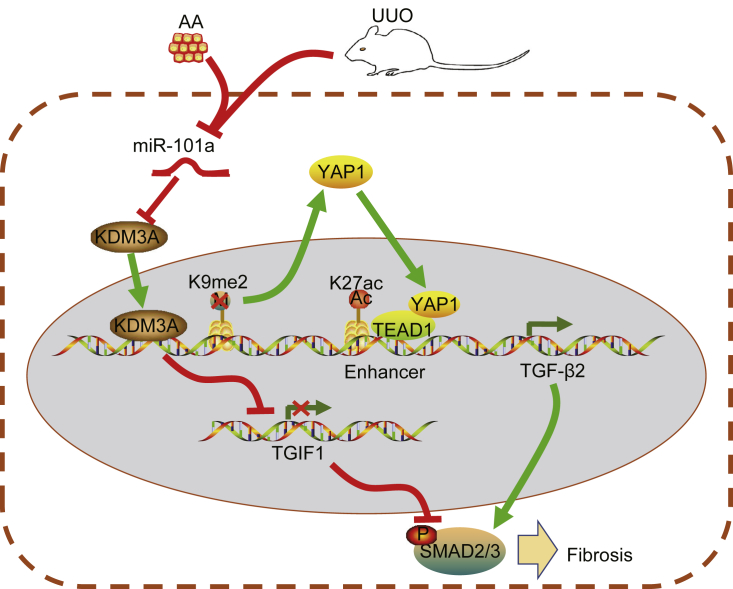
Molecular Mechanism Underlying the Regulatory Role of miR-101a in Alleviating Chronic Renal Fibrosis through Histone Demethylase KDM3A via Blockade of the YAP-TGF-β-Smad Signaling Pathway In AA-incubated HK2 cells and the UUO-induced chronic renal fibrosis mouse model, decreased expression of miR-101a and increased expression of KDM3A were detected. KDM3A may promote the expression of YAP1 and TGF-β2 through H3K9me2. In addition, KDM3A could regulate the binding of H3K27ac and TEAD1 to TGF-β2 and consequently promote the expression of TGF-β2. In addition, KDM3A could inhibit TGIF1 expression and its downstream signaling pathway. Therefore, results from the present study suggested that KDM3A could potentially promote renal fibrosis through the YAP-TGF-β-Smad signaling pathway.

Renal fibrosis is the unifying pathway eliciting the development of CKD.^18^ Emerging evidence has highlighted the function of miRNAs in renal fibrosis, which facilitates the process of early diagnosis and treatment of renal diseases.^19^ Therefore, the current study aimed to explore the roles of miR-101a in the progression of chronic renal fibrosis. The *in vivo* and *in vitro* findings suggested that upregulation of miR-101a could potentially inhibit chronic renal fibrosis via blockade of the YAP-TGF-β-Smad signaling pathway as a result of histone demethylase KDM3A (Figure 9).

Initially, chronic renal fibrosis tissues and cells presented with poorly expressed miR-101a. Based on a previous study, miR-101 is frequently downregulated in various diseases and cancers, such as RCC,^20^ as well as in cardiac tissues of rheumatic heart disease patients.^21^ miRNAs have been revealed to associate with renal development, homeostasis, as well as physiological functions in kidneys.^19^ The present study revealed that the upregulation of miR-101a could delay chronic renal fibrosis. Similarly, a previous study also reported that miR-101a could inhibit cardiac fibrosis produced following hypoxia.^9^ Interestingly, the combination of miR-101 and miR-494 has shown to markedly suppress cystic fibrosis by blocking cystic fibrosis transmembrane conductance regulator (CFTR) activity.^22^

Additionally, we found that KDM3A was upregulated in chronic renal fibrosis. RCC samples showed a notable higher KDM3A expression in comparison to normal adjacent kidney tissues.^10^ Results from a previous study suggested that the expression of KDM3A was elevated in chronic kidney disease.^23^ In the present study, results obtained from the combination of the online website (http://www.microrna.org/) and dual-luciferase reporter assay confirmed that KDM3A was a target gene of miR-101a and could be negatively regulated by miR-101a. Previous data have demonstrated that KDM3A may be the target gene of several miRNAs. For instance, KDM3A has been identified as a new miR-22-targeted gene, and its expression can be inhibited by miR-22, thus preventing Ewing sarcoma clonogenic and anchorage-independent cell growth.^24^

In addition, we uncovered that miR-101a could repress chronic renal fibrosis via inhibition of KDM3A expression. It was indicated by the diminished positive expression rate of Col1a1, fibronectin, and α-SMA proteins, as well as by the attenuated fibrosis degree of kidney tissues upon KDM3A silencing or overexpressed miR-101a *in vivo* and *in vitro*. The overexpression of miR-122 has the capacity of suppressing fibrosis-related genes, including Col1a1, fibronectin, and α-SMA, in hepatic stellate cells and fibroblasts.^25^ Inhibited expression of Col1a1, fibronectin, and α-SMA expression hinder renal fibrosis in UUO mice and NIH 3T3 fibroblasts.^26^ KDM3A can be able to regulate the activation of hepatic stellate cells and liver fibrosis via epigenetic regulation of PPARγ.^15^ Therefore, we concluded that miR-101a could potentially attenuate chronic renal fibrosis by suppressing KDM3A expression.

Another important finding was that KDM3A might promote the expression of YAP1 and TGF-β2 through H3K9me2 and, therefore, KDM3A could exert a regulatory role in the expression of TGF-β2 by regulating the binding of H3K27ac and TEAD1 to the enhancer of TGF-β2. Consistently, inhibition of H3K9me2 has been shown to remarkably stimulate YAP1 expression in colorectal cancer cells.^12^ TGF-β has been considered to be a leading regulator during the process of renal fibrosis owing to its ability to stimulate the accumulation of extracellular matrix proteins, thus resulting in kidney function impairment.^19^ Also, KDM3A has the potency to promote the binding of H3K27ac and TEAD1 to the enhancers of hippo target genes.^12^ KDM3A has been detected to downregulate the expression of TGIF1 in renal fibrosis. TGIF1 is a known multifunctional protein capable of repressing the activation of TGF-β signaling pathway due to its interaction with Smad2-Smad4 complexes.^17^ Since the TGF-β/Smad signaling pathway potentiates renal fibrosis and inflammation, it thus plays a critical role in chronic progressive kidney diseases.^27^ Knockdown of YAP/TAZ and TGF-β/Smad crosstalk may accelerate the anti-fibrotic activity of verteporfin.^13^ On the basis of the aforementioned evidence, it can be concluded that KDM3A could promote chronic renal fibrosis through activation of the YAP-TGF-β-Smad signaling pathway.

In summary, *in vitro* and *in vivo* experiments in the present study demonstrated that overexpression of miR-101a could suppress chronic renal fibrosis via histone demethylases KDM3A, along with the blockade of the YAP-TGF-β-Smad signaling pathway. However, future studies are recommended that employ specimens from chronic renal fibrosis-diagnosed patients for the in-depth investigation on the regulatory role of miR-101a in chronic renal fibrosis.

## Materials and Methods

### Ethics Statement

The Animal Care and Use Committee (ACUC) approved the current study, which was then conducted in accordance with National Institutes of Health guidelines. All measures were made to minimize the suffering of animals.

### Establishment of UUO-Induced Renal Fibrosis Mouse Model

A total of 160 C57BL/6 mice (age, 7 weeks old; weight, 23–28 g; Jackson Laboratory, Bar Harbor, ME, USA) were selected and raised on a 12-h light/12-h dark cycle, followed by undergoing UUO. Briefly, the mice were anesthetized by pentobarbital, and the left ureter was isolated from the surrounding tissues and subsequently double ligated. Next, the partial ureter between the two ligations was cut, and then 500 μL of sterile normal saline was injected into the abdominal cavity and the incision was closed. Eight mice that underwent similar surgical procedures yet without UUO were regarded as the sham-operated mice. In addition, 152 mice were injected with green fluorescent protein (GFP) lentivirus vector containing WT KDM3A (1 × 10^8^ plaque-forming units [PFU]/100 μL) or TGBβ2, pSIH1-H1-copGFP lentivirus vector containing KDM3A-specific short hairpin RNA (shRNA; 1 × 10^8^ PFU/100 μL), or miR-101a agomir via caudal vein twice every week, for a total of 6 weeks. The aforementioned lentivirus vector or agomir was constructed by Shanghai GenePharma (Shanghai, China). Agomir is based on chemically synthesized mimics, specially modified by chemical modification. It can be directly dissolved and used in animal experiments. The structure and use are different from mimics, but the effect on miRNA upregulation is the same. Thus, miR-101a-agomir was used for animal experiments and miR-101a mimic was used for cell experiments in the current study. The sequences of agomir-negative control (NC) and miR-101a-agomir were 5′-AAAAGAGACCGGUUCACUGUGA-3′ and 5′-UACAGUACUGUGAUAACUGAA-3′, respectively. The sequences of miR-101a mimic were 5′-UACAGUACUGUGAUAACUGAA-3′ (forward) and 5′-CAGUUAUCACAGUACUGUAUU-3′ (reverse), and those of mimic-NC were 5′-UUCUCCGAACGUGUCACGUUU-3′ (forward) and 5′-ACGUGACACGUUCGGAGAAUU-3′ (reverse).

A total of 136 mice were successfully modeled, with a success rate of 89.47%. These mice were then injected with lentiviruses expressing miR-101a-agomir, sh-KDM3A, miR-101a agomir + oe-KDM3A, oe-YAP, oe-TGBβ2, sh-KDM3A + oe-YAP1, sh-KDM3A + oe-TGF-β2, miR-101a agomir + oe-YAP1, miR-101a agomir + oe-TGF-β2, and miR-101a agomir + sh-TGIF1 as well as their corresponding controls. All of the mice were euthanized on the 14th day following UUO, and the kidneys were collected for subsequent experiments.

### Immunohistochemistry

The kidney tissues were first made into 5-μm-thick sections, followed by dewaxing and hydration. Following this, the tissue sections were immunostained with the following diluted antibodies attained from Abcam (Cambridge, UK): rabbit polyclonal antibody to Col1a1 (1:100, ab34710), rabbit polyclonal antibody to fibronectin (1:50, ab2413), rabbit monoclonal antibody to α-SMA (1:500, ab108424), and rabbit polyclonal antibody to phosphorylated (p-)Smad2/3 (1:100, sc-11769, UNIV Biotechnology, Shanghai, China). Next, the tissue sections were incubated with biotin-labeled secondary goat anti-rabbit immunoglobulin G (IgG) (1:500, ab97049, Abcam) for 30 min. Finally, five high-power visual fields were randomly selected and 100 cells were counted in each field under a light microscope.

### Masson’s Trichrome Staining

The nuclei of kidney tissue sections were stained with Weigert’s hematoxylin (G1142, Beijing Solarbio Science & Technology, Beijing, China) and Masson’s trichrome solution for 5 min, respectively. Next, the interstitial fibrosis and structure changes of tissues were observed under an optical microscope (DSX100, Olympus Optical, Tokyo, Japan). Collagen fibers appeared green or blue, nuclei appeared gray-black or gray-blue, while the muscle and cytoplasm of red blood cells were red.

### FISH

FISH was conducted by means of miRNA localization protocols as decribed.^28^ The fluorescence probes to miR-101a were from Exiqon (Woburn, MA, USA) and hybridized to mouse testis sections. All reagents and apparatus were treated with diethyl pyrocarbonate (DEPC). Hybridizations were next conducted at a temperature of 58°C for one night. Sections were stained with 4′,6-diamidino-2-phenylindole, and the *in situ* signal was visualized by confocal microscopy.

### Cell Treatment

Human renal tubular epithelial HK2 cells obtained from the American Type Culture Collection (ATCC, Manassas, VA, USA) were cultured in Dulbecco’s modified Eagle’s medium (DMEM; Sigma-Aldrich, St. Louis, MO, USA) containing 10% fetal bovine serum (Sigma-Aldrich) and 100 U/mL penicillin and streptomycin (Gibco, Grand Island, NY, USA). Next, the cells were prepared into suspension at a cell density of 2.5 × 10^4^ cells/mL, followed by inoculation onto a six-well plate (2 mL/well). Then, 2 × 10^6^ transduction units (TU) of corresponding lentivirus Polybrene (H9268, Sigma-Aldrich) with the final concentration of 8 μg/mL was added to 1 mL of serum-free medium containing 100 U/mL penicillin and streptomycin. After a 48-h period of transfection, puromycin (P8833, Sigma-Aldrich, St. Louis, MO, USA) was added to each well to screen the stably transfected cells. H3K9me2 inhibitor UNC0631 was used to treat HK2 cells as previously reported.^12^ Afterward, the HK2 cells underwent a treatment with AA (S9193, Selleck Chemicals, Houston, TX, USA) with a concentration of 5 μg/mL for a period of 48 h, after which AA was then washed away and the cells underwent further culture for a period of 24 h. As per the manufacturer’s instructions for Lipofectamine 2000 (Invitrogen, Carlsbad, CA, USA), the HK2 cells incubated with AA were further subjected with transfection with miR-101a mimic, oe-KDM3A, sh-KDM3A, miR-101a mimic + oe-KDM3A, oe-KDM3A H1120A, sh-KDM3A + vehicle (veh), sh-KDM3A + UNC0631, sh-YAP, sh-TGF-β2, sh-KDM3A + oe-YAP, sh-KDM3A + oe-TGF-β2, oe-TGIF1, miR-101a mimic + oe-YAP1, miR-101a mimic + oe-TGF-β2, and miR-101a mimic + sh-TGIF1 as well as their corresponding controls.

### RNA Isolation and Quantitation

Total RNA content extraction was first performed from tissues or cells by means of TRIzol (16096020, Thermo Fisher Scientific, Waltham, MA, USA). The extracted RNA was next reverse transcribed into complementary DNA (cDNA) with the use of a TaqMan miRNA reverse transcription kit (4366596, Thermo Fisher Scientific, Waltham, MA, USA) and a high-capacity cDNA reverse transcription kit (4368813, Thermo Fisher Scientific, Waltham, MA, USA). The expression of miR-101a was thereafter assessed with the use of a TaqMan miRNA assay kit (Applied Biosystems, Carlsbad, CA, USA) with the U6 gene as the internal reference. β-Actin was employed as the internal reference for the remaining genes. Primer sequences were next subjected to synthesis by Takara (Table 2). The fold changes were calculated using the 2^−ΔΔCt^ method.

**Table 2:** Primer Sequences for qRT-PCR

Gene	Sequences (5′→3′)
m-KDM3A	F: CGTTACTAAGAAAGACTTGAAGGTG
	R: CCAGGTTATGCTCTACTAAAAATGC
h-KDM3A	F: ATGCTGCAAAGGACACGG
	R: GAACTCCATACTCTTGATGAAGACG
m-miR-101a	F: GAGGGGTACAGTACTGATA
	R: TGCGTGTCGTGGAGTC
h-miR-101a	F: TCCCCCGGGCCAGAGGTT GTAACGTTGTCTAT
	R: GAAACCCAGCAGACAAAGCT TTGTTGCCTAACGAAC
m-U6	F: GCATGACGTCTGCTTTGGA
	R: CCACAATCATTCTGCCATCA
h-U6	F: CTCGCTTCGGCAGCACA
	R: AACGCTTCAGGAATTTGCGT
m-YAP1	F: CTGCGTGCAGAAATGCTACTG
	R: AGCCGTAGAGTAATGGTGGATAG
h-YAP1	F: CCTGATGGATGGGAACAAGC
	R: GCACTCTGACTGATTCTCTGG
m-TGF-β2	F: GGAGGTGATTTCCATCTACAA
	R: GGGAGATGGTAAGTCTTTGGGA
h-TGF-β2	F: CCCACATCTCCTGCTAATGT
	R: GCTGAGTGTCTGAACTCCAT
m-TGIF1	F: AGAGGCAATCTGCCCAAGG
	R: GGGATAGGCGTTGTATCTGTG
h-TGIF1	F: GCTGTCCCAGCAAACACACC
	R: TTCTCAGCATGTCAGGGAGGA
m-β-actin	F: ACGTGCCGCCTGGAGAAAC
	R: GTCCTCAGTGTAGCCCAAGATGC
h-β-actin	F: GTGAAGGTGACAGCAGTCGGTT
	R: GAAGTGGGGTGGCTTTTAGGA

qRT-PCR, quantitative reverse transcription polymerase chain reaction; m, mouse; h, human; KDM3A, lysine (K)-specific demethylase 3A; YAP1, Yes-associated protein 1; TGF-β2, transforming growth factor β2; TGIF1, TGFB-induced factor homeobox 1; F, forward; R, reverse.

### Western Blot Analysis

Western blot analysis was carried out as previously described^29^ with the following antibodies from Abcam: rabbit anti-Col1a1 primary antibody (ab34710, 1:1,000), rabbit anti-fibronectin primary antibody (ab45688, 1:1,000), rabbit anti-α-SMA primary antibody (ab108424, 1:1,000), rabbit anti-p-Smad2/3 primary antibody (ab63399, 1:1,000), rabbit anti-Smad2/3 primary antibody (ab202445, 1:1,000), rabbit anti-KDM3A primary antibody (ab106456, 1:1,000), mouse anti-β-actin primary antibody (ab8226, 1:1,000), and horseradish peroxidase-labeled secondary antibody (ab205719, 1:2,000). An image analysis system from Bio-Rad (Hercules, CA, USA) was employed for imaging, and Quantity One v4.6.2 software was employed for protein quantification analysis. The ratio of the gray value of the target band to β-actin represents the relative protein expression.

### Dual-Luciferase Reporter Assay

The target gene of miR-101a was analyzed. Gene fragments of the KDM3A 3′ untranslated region (3′ UTR) were artificially synthesized and subsequently introduced into pGL3-reporter (Promega, Madison, WI, USA) using endonuclease sites XhoI and BamHI. The complementary sequence mutation sites of seed sequences were designed on KDM3A-WT. Following restriction endonuclease, the target fragments were inserted into pGL3-reporter vector with T4 DNA ligase. The constructed luciferase reporter plasmids WT (pGL3-KDM3A-WT) and MUT (pGL3-KDM3A-MUT) were co-transfected into HEK293 cells (ATCC, Manassas, VA, USA) with miR-101a mimic or mimic-NC, respectively, followed by incubation in a 37°C incubator with 5% CO_2_ and saturated humidity. The culture medium was replaced with DMEM (Sigma-Aldrich, St. Louis, MO, USA) every 2–3 days. After a 48-h period of transfection, the cells were harvested and lysed. Luciferase activity was next detected on a luminometer TD-20/20 detector (E5311, Promega, Madison, WI, USA) by means of a Dual-Luciferase reporter assay system kit (Promega, Madison, WI, USA).

### ChIP

ChIP was performed with the use of an EZ-Magna ChIP kit (EMD Millipore, Billerica, MA, USA) as previously described.^30^ In brief, HK2 cells were first subjected to fixation with 4% paraformaldehyde and a 10-min period of incubation with glycine to allow the production of DNA-protein cross-linking. Then, the cells were washed twice with PBS and lysed with SDS lysis buffer (Upstate Biotechnology, Lake Placid, NY, USA). The lysates were then subjected to ultrasonication to produce the 200- to 300-bp chromatin fragments. After centrifugation at 15,000 × *g* for 10 min, the supernatants were diluted with ChIP dilution buffer (Upstate Biotechnology, Lake Placid, NY, USA) and were immunoprecipitated overnight with rabbit antibodies at 4°C. The antibodies used in the experiment include IgG (ab171870), KDM3A (ab91252), TEAD1 (ab133533), H3K9me2 (ab1220), H3K4me1 (ab8895), and H3K27ac (ab203953), all of which were purchased from Abcam. The normal anti-IgG antibody (2 μg, ab171870, Abcam) was used as a control of immunoprecipitation. The beads were washed in low-salt solution for 5 min at 4°C, and then washed twice in 1× Tris-Elhylene diamine tetraacetic acid (Upstate Biotechnology, Lake Placid, NY, USA) at room temperature for 2 min. The DNA was eluted from the beads, and serial dilutions of DNA (1:4, 1:20, 1:100, and 1:500) were analyzed using qRT-PCR to determine the precipitated DNA. The following primers were used: TGF-β2 enhancer primer, forward, 5′-CAGCCTTGATGAAAGGAAGC-3′, reverse, 5′-GGCAAGCAACTTCTCTCCTG-3′; TGF-β2 P1 promoter primer, forward, 5′-CGCCAAGGAGGTTTACAAAA-3′, reverse, 5′-CTCAGGGGATGGAAGTCAAA-3′; TGF-β2 P2 promoter primer, forward, 5′-CGCCAAGGAGGTTTACAAAA-3′, reverse, 5′-AAACCTCAGGGGATGGAAGT-3′; TGF-β2 P3 promoter primer, forward, 5′-TCCCTTCCCCCTAACAATTC-3′, reverse, 5′-CTGGTGGCCGACTAAAGAAC-3′; YAP1 P1 promoter primer, forward, 5′-CGTTTGAGGCGAGTTTCTGT-3′, reverse, 5′-CGCCTCCCCTTTCTCTTTAT-3′; YAP1 P2 promoter primer, forward, 5′-GAGGAAGGAAGAGCCGAGAG-3′, reverse, 5′-GCCTCAAACGCCAAAACTAA-3′; YAP1 P3 promoter primer, forward, 5′-AGAGGAAGGAAGAGCCGAGA-3′, reverse, 5′-GCCTCAAACGCCAAAACTAA-3′.

### Statistical Analysis

Statistical analyses were performed with the use of SPSS 21.0 software (IBM, Armonk, NY, USA). Measurement data were summarized as a form of mean ± standard deviation. Data in adherence to normal distribution and homogeneity of variance between two groups were compared using the unpaired t test, whereas variance among multiple groups was compared by one-way analysis of variance (ANOVA), followed by Tukey’s multiple comparisons post-test. Statistical significance was reflected by a value of p < 0.05.

## Author Contributions

H.D. and Y.X. designed the study. N.J. and Y.X. collated the data, carried out data analyses, and produced the initial draft of the manuscript. Y.X. and H.D. contributed to drafting the manuscript. All authors have read and approved the final submitted manuscript.

## Conflicts of Interest

The authors declare no competing interests.
